# Should deciduous teeth be preserved in adult patients? How about stem cells? Is it reasonable to preserve them?

**DOI:** 10.1590/2177-6709.21.2.015-027.oin

**Published:** 2016

**Authors:** Alberto Consolaro

**Affiliations:** 1 Full professor, Universidade de São Paulo (USP), Undergraduate program, Bauru, São Paulo, Brazil and Universidade de São Paulo (USP), Graduate program, Ribeirão Preto, São Paulo, Brazil.

**Keywords:** Deciduous teeth, Root resorption, Stem cells, Orthodontic movement, Apoptosis

## Abstract

When seeking orthodontic treatment, many adolescents and adult patients present with deciduous teeth. Naturally, deciduous teeth will inevitably undergo exfoliation at the expected time or at a later time. Apoptosis is the biological trigger of root resorption. In adult patients, deciduous teeth should not be preserved, as they promote: infraocclusion, traumatic occlusion, occlusal trauma, diastemata and size as well as morphology discrepancy malocclusion. Orthodontic movement speeds root resorption up, and so do restoring or recontouring deciduous teeth in order to establish esthetics and function. Deciduous teeth cells are dying as a result of apoptosis, and their regeneration potential, which allows them to act as stem cells, is limited. On the contrary, adult teeth cells have a greater proliferative potential. All kinds of stem cell therapies are laboratory investigative non authorized trials.

## Resumo

Muitos adolescentes e adultos, ao procurar pelo tratamento ortodôntico, apresentam dentes decíduos persistentes. Naturalmente, os dentes decíduos ou se esfoliam na época esperada ou mais tardiamente, de forma inevitável. A apoptose é o gatilho biológico da rizólise. Em adultos, os dentes decíduos não devem ser preservados, pois promovem: infraoclusão, oclusão traumática, trauma oclusal, além de diastemas e má oclusão por discrepância de tamanho e morfologia. O movimento ortodôntico acelera o processo de rizólise, assim como restaurar ou reanatomizar dentes decíduos para inseri-los em uma estética e função. As células dos dentes decíduos estão morrendo por apoptose e seu potencial regenerativo para atuarem como células-tronco tem limitações, ao contrário das células de dentes adultos, que têm maior potencial proliferativo. Todas as terapias com células-tronco ainda são laboratoriais e se enquadram como ensaios investigativos não autorizados.

Approximately 25% of the overall population has some kind of partial anodontia, which implies in the presence of deciduous teeth in adult patients' mouth after the expected time for exfoliation. Based on the frequency with which dental care professionals seek advice and aim at clarifying doubts on a number of aspects related to deciduous teeth, we offered to have articles published on the theme, so as to lay the basis for clinicians' attitude and protocols when treating patients and interacting with colleagues involved with relevant treatment plans. For instance, when seeking orthodontic treatment, many adolescents and adult patients present with deciduous teeth. In this scenario, one might ask: Should we extract, preserve, accommodate or move those teeth? Should we perform space closure or place an osseointegrated implant? 

In addition, in the last few years, much information has been disclosed - whether commercially or not, and mostly in the lay media rather than within the scientific community - on the use of deciduous teeth cells as a potential source and storage of stem cells in favor of future therapeutic application. Regardless of their specialty, dental care professionals must be well grounded in order to be able to judge, put into practice and make decisions on the subject. Deciduous teeth are a matter of concern of all dental specialties, even though it has been generally and erroneously believed that it is a theme limited to Pediatric Dentistry investigation.

## WHAT DOES THE TERM "DECIDUOUS TEETH" MEAN? 

Deciduous is an adjective that describes something that falls off, it is caducous or confined. Deciduous makes reference to the parts of plants or animals that fall off before the usual time at a certain stage of development in the life cycle. Caducous refers to something that is about to fall off, something that has lost the strength and vigor or something that has been annulled and, therefore, has a limited life span. In Latin, the verb *decidere* means to fall off, to perish or to die.

Deciduous teeth are those which descend, fall off or become caducous. They are primary, temporary teeth continuing for a limited amount of time within a child's mouth. When they become soft and fall off, those teeth undergo exfoliation, as it happens with flower petals and leaves. Deciduous teeth are preferably termed "temporary" or "primary" by Anglo-Saxon authors, as they consider "deciduous" a popular and rather lay term. Nevertheless, it perfectly describes the conditions of such teeth. 

Physiological tooth resorption only occurs in deciduous teeth. Permanent teeth do not primarily take part in root resorption which is set up in all deciduous teeth, with or without the presence of permanent successors. Permanent teeth do not undergo exfoliation, but speed the process of root resorption up as they embrace deciduous teeth in their course of eruption. This is a result of the intense concentration of bone resorption and hard tissue mediators found in the pericoronal follicles.

## WHAT TRIGGERS ROOT RESORPTION IS APOPTOSIS!

A fully developed deciduous tooth has mineralized hard tissues (enamel, dentin, cementum and bundle bone) as well as soft tissues (pulp, periodontal ligament and gingiva). Should mineralized tissues be exposed to the connective tissue, they not only undergo resorption by clastic cells combined with other cells, but also disappear, while the enamel undergoes exfoliation within the oral environment and is sometimes carried by saliva via the gastrointestinal tract.

But what happens to soft tissue cells of deciduous teeth? Exfoliation of deciduous teeth is somewhat already part of human nature. During development and growth, that process happens without any symptoms, it is natural and physiological. Thymus and thyroglossal duct undergo the same process, disappearing as we reach adulthood.

**Figure 1:**
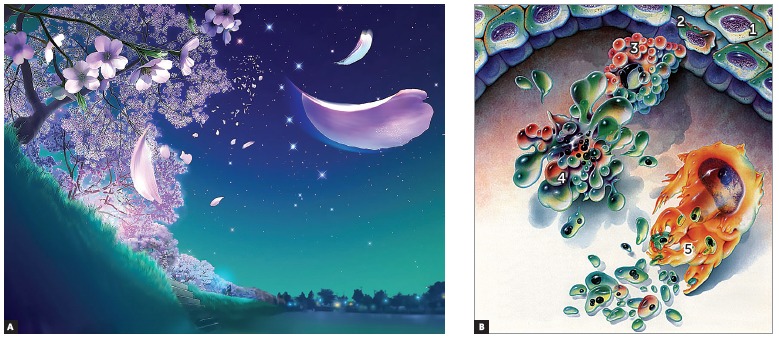
From the Greek, apoptosis means defoliate and plucking of petals typical of the fall, as shown in A. In B, note: a normal cell (1); cell at the early stages of apoptosis, shrinking (2); condensed chromatin with small lumps of cytoplasm about to be released (3); nuclear and cytoplasmic fragmentation, forming the first apoptotic corpuscles (4) phagocytized by neighboring cells and macrophages (5) (B = modified from Duke et al.^7^, 1996).

Once a deciduous tooth has fully developed, its cells are genetically programmed to release or derepress gene *p53* which, in combination with other genes, controls the biochemical process of cell collapse. The cytoskeleton, the inner structure that holds the cell together, will be broken and destroyed by active enzymes, and the cell will undergo outer, severe, gradual shrinkage (Fig 1), as if it were a passion fruit forgotten in a fruit bowl. 

Meanwhile, protein synthesis becomes limited and increasingly restricted. The chromatin will be arranged in disorganized lumps. The cell will have a tendency to break into several fragments or corpuscles, each one carrying a piece of cell enclosed by cell membrane. This process is limited to a few cells - here and there - without releasing enzymes or without "bothering" neighboring cells or damaging tissue components. Whenever neighboring cells recognize any fragments, they aid the macrophages to phagocytize them. 

The process of asymptomatic, natural cell elimination is best known as apoptosis, a Greek term for the loss of leaves or petals, an analogy to trees and flowers during the fall season (Fig 1): similarly to "shooting leaves or flowers", cells lose pieces, or "petals", which tend to detach from one another. 

Apoptosis is also known as programmed cell death or cell suicide. In the odontogenesis of rats and cats, as well as in cats' and humans' deciduous teeth, apoptosis was first seen as a theme of Masters and Doctorate thesis and dissertation written by Lourenço^3^ ^,^ ^4^.

Apoptosis is also part of other processes that occur in the human body, such as the elimination of cells and tissues from between fingers, thus setting them apart; in median cell cords, thus originating ducts, other hollow structures and channels through which secretion flows. Atypical cells appear on a daily basis among 10 trillion proliferating cells and might become malignant cells in the future. They are eliminated early by means of apoptosis induced by immune system mediators and cells or by means of mechanisms happening inside the cells, whenever gene *p53* has been derepressed. 

**Figure 2:**
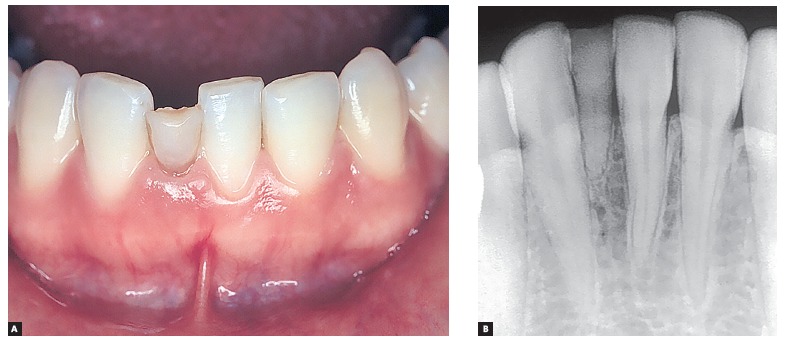
Deciduous tooth found in and adult patient's mouth, revealing infraocclusion and tooth root with preserved structure, but also revealing irregular areas of surface resorption, with imprecise lamina dura delimitation and periodontal space.

**Figure 3:**
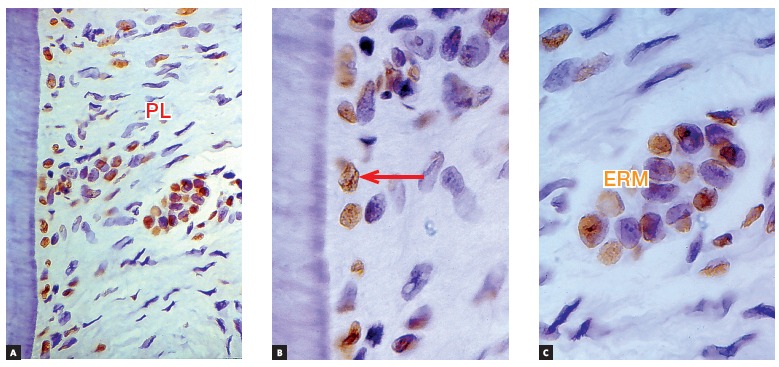
Root surface and periodontal ligament (PL) of a human deciduous tooth immunohistochemically marked to identify cell proteins directly associated with apoptosis (TUNEL method). Apoptosis seems clear in cementoblasts (arrow) and Epithelial Rests of Malassez (ERM). (A = 10X, B = 40X and C = 100X).

**Figure 4:**
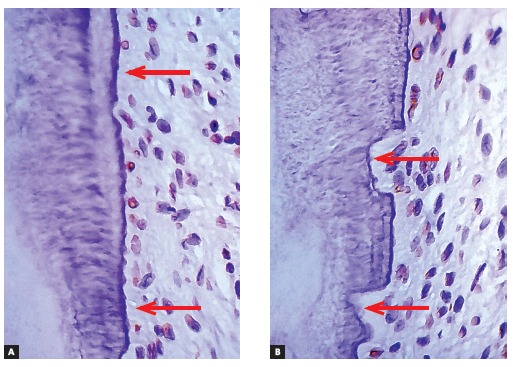
Root surface and periodontal ligament of a human deciduous tooth immunohistochemically marked to reveal cementoblasts under apoptosis (TUNEL method) already absent in a few regions (arrows) where root resorption has already been initiated (arrows in B) (A =10X, B = 40X).

In deciduous teeth, apoptosis causes pulp and periodontium cells to disappear; thus exposing local and gradually both inner and outer surfaces of mineralized tissues. This process occurs throughout the entire root and might take months or even years (Figs 2, 3 and 4). Whenever mineralized tooth surfaces are exposed, without being protected or enclosed by cementoblasts, regardless of being of deciduous or permanent teeth, they tend to be naturally colonized by clastic cells, thus setting up areas of root resorption (Fig 4).

Should the exposed area be small or medium, permanent teeth subjected to trauma will undergo root resorption for a short period of time; however, if the area has not been contaminated by bacteria and if post-trauma inflammation ceases, neighboring cementoblasts will soon enclose the root surface, thus making the whole process stop. For tooth resorption to occur without interruption, there must be stimuli provided by mediators of inflammatory processes and tissue stress resulting from secondary contamination or occlusal trauma.^2^

In deciduous teeth, although apoptosis leaves a number of root surface micro areas exposed (Fig 4), over which clastic cells colonize, those areas tend to be very slow at reabsorbing hard dental tissues. This slow pace is determined by the extremely low level of local mediators stimulating clastic cells to act, since inflammation or local stress are absent and, therefore, do not allow mediators to build up in large amounts. 

The small amount or the absence of mediators do not provide stimuli for fast resorption of deciduous tooth roots. Even so, neighboring cementoblasts cannot repopulate denuded root areas, as they are genetically limited cells - most of which are undergoing pre-apoptosis or full apoptosis processes, without being capable of proliferating significantly.

**Figure 5:**
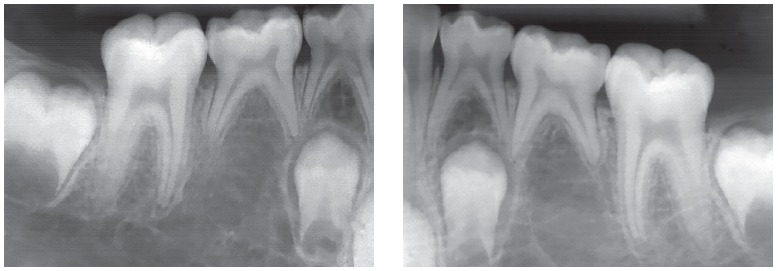
Deciduous molars in a patient with partial anodontia of second premolars. At this stage, the case must be well planned with a combination of knowledge and experience shared by pediatric dentists, implant dentists and orthodontists, without postponing precise clinical decisions.

In short: apoptosis of cementoblasts (external) and odontoblasts (internal) is the biological trigger of root resorption. Root resorption occurs in both surfaces of a deciduous tooth: inner and outer. Root resorption is triggered as soon as a deciduous tooth has fully developed, and does not rely on permanent successors and their tissues (Fig 5).

## THE ROLE PLAYED BY THE PERICORONAL FOLLICLE IN ROOT RESORPTION

Biological phenomena are mediated by chemical substances via cell surface receptors. Some cytokines as well as growth factors take part in inducing apoptosis; while others speed bone and mineralized tissue resorption up. With deciduous teeth, this process is triggered by mediators present in the pericoronal follicles of neighboring permanent teeth.

**Figure 6:**
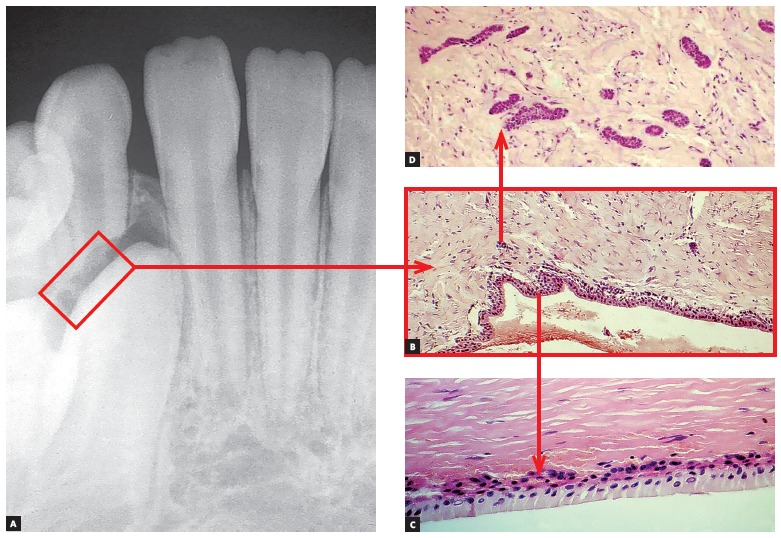
The pericoronal follicle is found at the pericoronal space. The former is made up of reduced enamel epithelium and of fibrous connective tissue (B). In C, a close-up of the reduced enamel epithelium (arrow). In D, a high number of clusters of dental lamina remaining odontogenic epithelial cells is found in the follicular connective tissue (arrow( (HE; B = 10X; C and D = 40X).

EGF (or Epidermal Growth Factor) is a mediator that stands out for providing local stimuli for mineralized tissue resorptive processes. It is secreted by and present in the epithelia, being found in higher concentrations in neighboring tissues. The pericoronal follicle or pericoronal membrane (Fig 6) is an anatomical structure rich in EGF due to having a large amount of epithelia, such as the reduced enamel epithelium and the clusters of epithelial cells remaining from the dental lamina (Fig 6). The EGF controls the activity of other mediators also present in pericoronal follicles.

One of the surfaces of a deciduous tooth is usually facing and found to be near the pericoronal follicle of a permanent tooth (Fig 6). On this surface, root resorption is significantly sped up by the larger amount of mediators inducing and accelerating apoptosis as well as mediators inducing the activity of clasts, such as EGF, among others.

Even after the usual time expected for exfoliation to happen, any deciduous tooth will inevitably have root resorption triggered - regardless of the existence or not of a permanent successor near it. Apoptosis and resorption of deciduous teeth do not rely on the presence of a permanent tooth and its pericoronal follicle. However, should the pericoronal follicle be near a deciduous tooth, it will speed the process of root resorption up, especially on the tooth surfaces found near or facing the permanent tooth.

**Figure 7:**
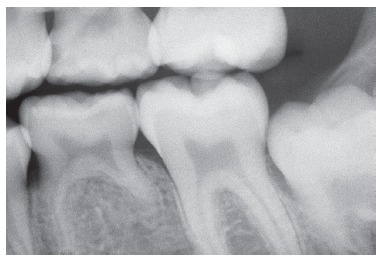
Adult deciduous tooth with replacement tooth resorption after alveolodental ankylosis.

**Figure 8:**
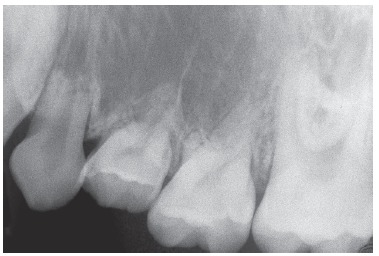
Root resorption, alveolodental ankylosis and replacement resorption occur in deciduous teeth, regardless of the presence of permanent successors, even if caused by anodontia of a group of teeth.

**Figure 9:**
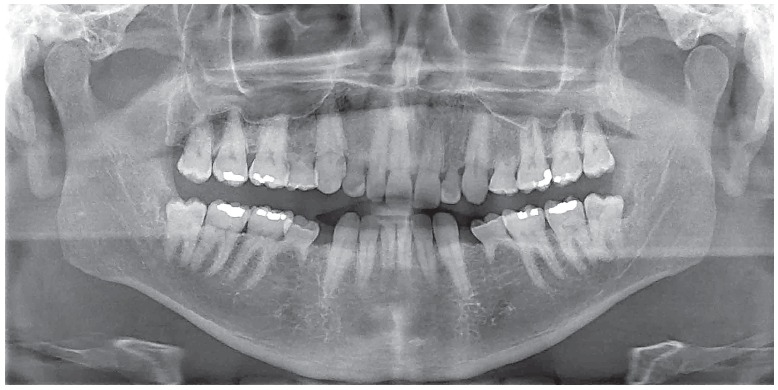
Adult deciduous teeth - in cases with multiple teeth affected by partial anodontia - associated with irregular loses over time lead to severe occlusal disharmony. Whenever carefully diagnosed and planned, the process should be avoided, so as to preserve spaces and/or move teeth properly in order to restore esthetics and function during adulthood.

**Figure 10:**
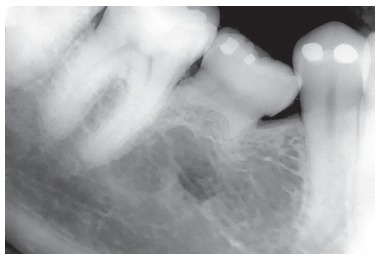
Adult deciduous teeth infraocclusion and size discrepancy among permanent and deciduous teeth lead to displacement and irregular space closure, with tipping of neighboring teeth and occlusal interference. Note that the deciduous tooth presents with replacement tooth resorption.

**Figure 11:**
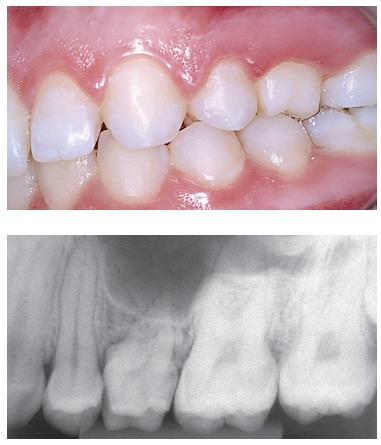
Occasionally, and in a special manner, some cases of adult deciduous teeth are temporarily solved with wear and movement of teeth into the space aimed at their permanent successor. Nevertheless, it should be highlighted that those are temporary solutions which might not always be the best option to patients.

A deciduous tooth without a permanent successor might, therefore, remain in one's jaw for many years (Figs 7 to 11). Its root resorption, however, began at the time its formation was complete. Should a deciduous tooth remain in one's jaw for many years without undergoing exfoliation, its root resorption is found to be too slow, particularly due to absence of mediators responsible for speeding the process up and of which source usually is the pericoronal follicle of permanent teeth.

## EPITHELIAL RESTS OF MALASSEZ IN DECIDUOUS TEETH DIE AS A RESULT OF APOPTOSIS: THIS IS THE REASON BEHIND ANKYLOSIS, REPLACEMENT RESORPTION AND SUBSEQUENT INFRAOCCLUSION!

Deciduous teeth undergo exfoliation thanks to cell death occurring as a result of apoptosis in their cementoblasts and odontoblasts, thus denuding the mineralized portion of the root and attracting clasts (Figs 3, 4). This process of root resorption is slow and takes place in the entire root surface due to lack of enough mediators necessary to speed the process up. 

Root resorption is sped up and becomes unidirectional when it occurs near the pericoronal follicle of a permanent tooth, as the latter counts with a significant amount of EGF as well as other mediators that trigger bone resorption. Pericoronal follicle mediators are responsible for exfoliation of deciduous teeth via root resorption and, at the same time, by bone resorption leading to eruption. 

The Epithelial Rests of Malassez are responsible for keeping the periodontal space unchanged, as they release EGF without interruption. Apoptosis also affects Epithelial Rests of Malassez cells (Fig 3) in such a way that, within 6 to 12 months after the usual time expected for exfoliation, alveolodental ankylosis is expected to happen. 

Should the Epithelial Rests of Malassez be absent in the ongoing process of bone remodeling, the bundle or alveolar bone gradually takes over the periodontal space. As a result of ankylosis, bone cells will gradually reabsorb bone and root, while irregularly taking turns with bone neoformation. This occurs in a such a way that the deciduous root is replaced by bone (Figs 7 to 10). Replacement tooth resorption in deciduous teeth is an expected evolution of cases in which exfoliation did not occur within the usual expected time*.*

It might sound a paradox, but ankylosis and replacement resorption of deciduous teeth induce infraocclusion, thus freeing the tooth from receiving occlusal load (Figs 2 to 10). Without occlusal trauma induced by overload resulting from adolescent and adult occlusion, the deciduous tooth subjected to ankylosis and undergoing replacement resorption will not have the amount of inflammatory mediators responsible for speeding the process of root resorption up increased, and the tooth shall remain longer at its original site, since most of the times the aforementioned process occurs due to absence of a permanent successor.

## WHY SHOULDN'T A DECIDUOUS TOOTH REMAIN IN AN ADULT PATIENT'S MOUTH? WHY SHOULDN'T IT BE USED FOR PROSTHETIC REPLACEMENT?

### 1) Infraocclusion, traumatic occlusion, occlusal trauma and induced accelerated exfoliation

Occlusion will always be considered an overload to adult patients' deciduous teeth. In occlusal trauma, inflammatory mediators build up, many of which will speed bone turnover up locally; sometimes providing stimuli for bone neoformation, sometimes providing stimuli for bone resorption. Whenever bone turnover speeds up, the rate of root structure loss increases, thus slowly inducing the tooth to remain attached to the underlying bone only by the cervical third of the crown.

This process rarely occurs because, from the beginning, ankylosis leads the tooth to infraocclusion, thereby preventing the deciduous tooth from being damaged by occlusal trauma resulting from traumatic occlusion.

Clinically, should prosthetic replacement be carried out in a deciduous tooth under the aforementioned conditions, with a view to restoring occlusion, the tooth crown will be the only structure found a month later. Occlusal overload will be established and occlusal trauma will speed the process of replacement resorption up (Figs 7 to 10).

This is the main reason why we should never trust the structural stability of a deciduous tooth and perform prosthetic or crown replacement with a view to apparently restoring esthetics and function. 

Such a maneuver might be useful as a therapeutic strategy employed to remove an affected tooth without the need for open surgery with bone cavities that might cause sequelae at the bone and gingival levels. One simple artifact used to have occlusion restored will be enough to trigger the accelerated process of root loss. 

### 2) Diastemata and malocclusion caused by size and morphology discrepancy 

Size and morphology of deciduous teeth are incompatible with those of permanent teeth. Should deciduous teeth remain in one's dental arch, with consequent late tooth loss, this will lead to discrepancies that result in excess or lack of space needed to restore the site with teeth equal in size to permanent teeth, or in cases of implant placement or orthodontic movement. In general, induced infraocclusion promotes displacement of neighboring permanent teeth distally and mesially, which results in occlusal interference (Fig 10). Size discrepancy contributes to the formation not only of diastemata, but also of undesired food impaction.

## AFTER ALL, SHOULD WE MOVE OR SHOULD WE NOT MOVE DECIDUOUS TEETH?

### a) With permanent teeth, what happens is the following: 

By compressing the periodontal ligament (which is 0.25-mm thick and has 50% of its total volume made up of blood vessels), stress and/or inflammation are induced. Both of these processes are characterized by local accumulation of mediators that provide stimuli for bone resorption on the periodontal surface of the alveolar bone.

Mediators such as cytokines, growth factors and prostaglandins provide stimuli for local bone resorption because they are at the same site, they attach to or interact with membrane receptors in osteoblasts, macrophages and clasts - the set of cells known as BMU, or bone modeling units. 

Cementoblasts covering the root surface of permanent teeth have no receptors for local or systemic mediators of bone resorption. Thus, the tooth moves in between bone structures, inducing resorption and remodeling periodontal tissues without causing root resorption. 

In summary, permanent teeth have their roots preserved due to the presence of cementoblasts destitute of receptors for resorption mediators: *cementoblasts protect the roots against tooth resorption.* *^1^* Mediators are present, but they do not interact with cementoblasts, they interact only with osteoblasts and osteoblast-related cells. 

That is the reason why teeth do not undergo root resorption when forces do not fully compress periodontal ligament vessels. Whenever movement is induced by extremely concentrated forces (and, for this reason, intense), cementoblasts might die as a result of anoxia. Additionally, denuded root surfaces will be subjected to resorption, even if temporarily.

### b) Orthodontic movement of deciduous teeth: Why not? 

In the orthodontic and/or orthopedic context, forces of any nature applied to the periodontal ligament of deciduous teeth will trigger the same type of stress and inflammation found in the periodontal ligament. Likewise, the same mediators will accumulate and bone resorption will occur on the alveolar bone periodontal surface. 

Nevertheless, once bone resorption mediators build up on the periodontal ligament, which is compressed, under stress and/or subjected to inflammation, osteoblasts, clasts and macrophages - arranged in the form of BMU - will also be stimulated to attach to the exposed root surface of the deciduous tooth.

A number of sites on deciduous tooth root surfaces no longer have the "coating" of cementoblasts which have died as a result of apoptosis. Whenever mineralized structures are directly exposed to the connective tissue, they attract or promote chemotaxis for the clasts. With orthodontic movement underway, more clasts will set up as a result of stimuli provided by bone resorption mediators: root resorption will be further sped up!

Root resorption of deciduous teeth is expected to speed up when those teeth are subjected to orthodontic movement. That is inherent to the process. Whenever a physiological structure such as the pericoronal follicle of a permanent tooth, surrounded by bone resorption mediators, approaches a deciduous root with areas lacking cementoblasts, root resorption is sped up. Likewise, whenever orthodontic movement is started, the deciduous tooth periodontal ligament will have a high local concentration of mineralized tissues resorption mediators on both surfaces: bone and root.

Should there be a need or an opportunity for orthodontic movement of deciduous teeth or the need to use them as anchorage units to move other teeth, or even for orthopedic purposes, one should be aware that: root resorption will be sped up and, as a result, the deciduous tooth will be subjected to exfoliation earlier. 

Clinicians, orthodontists or orthopedists assessing cases of deciduous teeth involved with orthodontic movement and/or anchorage planning (Figs 8, 9 and 10) should ponder the following: Is the clinical benefit provided to the patient relevant enough so as to be worth the risk of being subjected to early, inconvenient root resorption?

It should be determined that forces acting over deciduous teeth do not act orthopedically, expanding the arch so as to provide more space for permanent teeth, neither prevent cross bite and other future occlusal problems. Deciduous teeth might be used as anchorage units, as it is the case with expander appliances; whenever keeping those teeth is not of great importance for the next two to three months; or, especially, when deciduous teeth have been subjected to alveolodental ankylosis, with or without replacement resorption. 

Orthodontic movement of deciduous teeth does not prevent future occlusal problems affecting permanent teeth, except in a few cases, it rather speeds root resorption of deciduous teeth up with unpredictable consequences.

## SHOULD DECIDUOUS TEETH BE RECONTOURED AS IF THEY WERE PERMANENT TEETH? NO!

A very common clinical question in cases of deciduous teeth found in adult patients refers to the potential for increasing the clinical crown via prosthetic rehabilitation for esthetic or functional purposes, since they will be part of the occlusal plane. 

In a deciduous tooth, adult occlusion implies forces acting over the deciduous periodontal ligament which are, therefore, excessive and traumatic, thereby causing cellular stress and local inflammation (Fig 11). The increased amount of mediators at the site will speed root resorption up and, within a few weeks or months, the deciduous tooth in question will be subjected to exfoliation. 

The same line of thought applies to the potential for orthodontic movement of deciduous teeth in adult patients: there will be cellular stress and inflammation, with an increased amount of mediators at the site and accelerated root resorption. In no time, this tooth will be subjected to exfoliation. 

In short, one should not plan and "believe in" restorative and orthodontic procedures in deciduous teeth remaining in adult patients. Should a remaining deciduous tooth in an adult patient require restoration, it should not be reinserted into the occlusal plane, particularly if the goal is to keep the tooth as long as possible in the patient's mouth. 

Should there be a need for pulpotomy or pulpectomy, bacterial infection must be avoided at all costs; bacterial by-products should not spread beyond the dentin pulp surface, as it would induce inflammation of the periodontium while providing stimuli to speed root resorption up (which is already underway, but slow). Intrapulpal matter to be used must be the least aggressive and infiltrative possible at the dental structures, so as to prevent not only periodontal tissues from being damaged, but also root resorption from speeding up.

## WHAT SHOULD BE DONE IN THE PRESENCE OF ANODONTIA? SHOULD IT BE KEPT UNCHANGED? SHOULD RECONTOURING BE CARRIED OUT? OR SHOULD OCCLUSION BE RESTORED? HOW ABOUT EXTRACTION, IMPLANT PLACEMENT OR ORTHODONTIC PROCEDURES? HOW AND WHEN?

All procedures have an interesting, fruitful side. Root resorption of remaining deciduous teeth in adult patients is not an exception. Despite being subjected to slow root resorption due to absence of a permanent successor, when those teeth remain in one's mouth longer than expected after exfoliation, the epithelial rests of Malassez are also subjected to apoptosis. Without the epithelial rests of Malassez, the bone will naturally approach the tooth and allow alveolodental ankylosis to happen. This is a common finding in the orthodontic office. 

In addition to being smaller, the remaining deciduous tooth of an adult patient also tends to be in infraocclusion, as it has been subjected to ankylosis. In those cases, surgical removal of this tooth will cause loss at the bone level and, from a gingival, esthetic standpoint, hinder implant placement and orthodontic movement of neighboring teeth to the extraction site. 

A potential alternative taking advantage of biological knowledge to gain clinical benefits is composite resin placement on the occlusal surface of remaining deciduous teeth of adult patients with a view to including them into patient's occlusal plane. With occlusal trauma represented by masticatory load performed by an adult, mediator built up at the deciduous periodontal ligament as a result of cellular stress and inflammation will speed root resorption up. Within a few weeks, all that will be left is the deciduous tooth crown which can be easily removed without loss at the bone level and without further surgical procedures.

Apoptosis of the epithelial rests of Malassez, the resulting alveolodental ankylosis of deciduous teeth remaining in adult patients and consequent infraocclusion (occurring with a view to avoiding overload of periodontal tissues) might be understood as another wonderful, puzzling manner found by nature as a way to compensate for things: keeping a deciduous tooth in one's mouth longer than expected, in the event of partial anodontia of permanent successors! 

Presently, implant-supported prostheses and orthodontic movement might be performed for space closure. Clinically, professional intervention will improve anodontic patients' conditions. Nevertheless, nature did count on the progress of Dentistry at the time it idealized the human body!

Partial anodontia of permanent teeth affects, on average, 25% of the overall population. Those cases must be diagnosed as soon as possible at an early age, and the child's parents or guardians must be immediately informed and called upon to make a decision.

Pediatric dentists, implant dentists and orthodontists must work together, study the case regarding its prospect of craniomandibular growth, and come up with a follow-up schedule in order to decide whether they will keep space for future implant placement, to be performed as soon as the patient reaches adulthood, or whether the deciduous tooth will be extracted, with subsequent orthodontic space closure.

Diagnosis and planning must not be postponed (Figs 8, ^9^), they must be followed, cleared up and have their steps consistently applied at the scheduled time. One should not encourage deciduous teeth to be kept in an adult patient's mouth, as this will typically lead to unsatisfactory, late consequences. Additionally, there are no benefits to be gained if they are kept in an adult's mouth for an indefinite period of time.

## HOW ABOUT USING DECIDUOUS TEETH AS A SOURCE OF STEM CELLS: IS IT REASONABLE TO KEEP A DECIDUOUS TOOTH IN ONE'S ARCH FOR THAT PURPOSE?

As soon as deciduous teeth are fully developed, their cells immunohistochemically reveal that they are gradually dying due to apoptosis. Apoptosis is a process of cell death controlled by the cells themselves, resulting from dysregulation of *p53*, which initiates a series of biochemical intracellular phenomena that result in cytoskeleton breakage and degradation of cell nucleus and membrane. 

Within a few hours or days, that cell in which gene *p53* has been derepressed shrinks and begins to lose fragments, as if it were a flower that loses its petals or a tree that loses its leaves. As odontoblasts and cementoblasts die here and there, they leave deciduous teeth surfaces with small, numerous, increasing cell-free areas (Figs 3 and 4). Odontoblasts and cementoblasts protect odontogenic mineralized tissue surfaces against clasts, typical cells responsible for mineralized tissue resorption, regardless of their nature. 

The natural future of all cells of which deciduous teeth are made up is apoptosis, as a means of elimination!

At surface and cell-free areas subjected to apoptosis, the cementum and dentin areas will receive and have clasts attached, thereby inducing root resorption. This occurs regardless of the region of the root, whether apical, middle or cervical third. Indeed, root resorption is initially triggered, initiated, induced or provided by apoptosis of cementoblasts and odontoblasts of deciduous teeth.

Apoptosis is one of the most elegant and impressive mechanisms performed by the human body to eliminate undesirable cells, such as those which have reached their functional peak, have accomplished a specific mission or have become rebellious or defective and, therefore, are willing to attain autonomy and, whenever surviving, create specific, cancerous clones. Nearly all cell tissues are subjected to apoptosis; without it, we would have a much briefer life.

Although root resorption occurs simultaneously in all root surfaces and regions, including the inner surface, it is usually sped up and directed towards a few specific areas and situations as a result of being near a permanent successor. The pericoronal follicle of a permanent tooth is rich in a growth factor pertaining to the epithelia (Fig 6), including the reduced enamel epithelium and the clusters of epithelial cells remaining from the dental lamina present in its connective tissue.

The reduced enamel epithelium, which has already formed the enamel, is now steadily attached to the enamel, nurtured by a capsular connective tissue. Together, they form the pericoronal follicle. The major structure responsible for tooth eruption is the pericoronal follicle, as it is rich in EGF (epithelial growth factor), a growth factor that induces epithelial cell proliferation with a view to keeping tissue structure, as it is constantly renewed. Meanwhile, many EGF molecules act over the surrounding bone tissue, thereby inducing pericoronal bone resorption and digging way for the emergence of the tooth into the oral cavity.

While the pericoronal follicle, along with its mediators "controlled" by the EGF, promote pericoronal bone resorption in the course of tooth eruption, they speed up slow root resorption when they approach a deciduous tooth. As a result, resorption plans of deciduous roots are established towards the region where the permanent successor is. 

Follicular tissue will always be present between a permanent successor and a deciduous tooth when they are near one another. On one side, it will adhere to the enamel through the reduced enamel epithelium; whereas on the other side, it will adhere to the connective tissue rich in clasts, on the interface with the deciduous tooth surface. Root resorption is not induced by the presence of a permanent successor, but the later is indeed responsible for speeding it up and moving it towards a single direction!

## HOW ABOUT STORING DECIDUOUS TOOTH STEM CELLS?

In order to raise funds, scientists have to convince referees that their research projects are worth it. Those referees are usually university professors who also rely on the same type of fund provided by government agencies and private companies. Thus, competition is tough. A worldwide common means of putting pressure on referees is by anticipating projects or preliminary outcomes of initial trials and disclosing potential applications and benefits to the media, thus producing "truths and/or needs." 

An example of such pressure posed by the media is stem cells. Ordinary people believe that stem cells can be routinely used in patients and are a treatment modality employed in Medicine. There are no countries in the world allowing any stem-cell therapeutic protocol to be used in patients. All outcomes and potential benefits disclosed are either experimental or authorized trials, but they do not consist of a treatment protocol.

In clinical practice, stem cells represent hope. The risks patients might be exposed to still need to be further investigated. For this reason, protocols cannot be established yet. Among such risks, there is the growth of tumors resulting from stem cells implanted in one's organism. Despite all restrictions and limitations, some people and some companies spend and make money with storage of cord blood and bone marrow stem cells. Lately, cells collected from exfoliated deciduous teeth, which are replaced by permanent teeth, have also been stored. 

None of stem cells companies and banks can guarantee that those cells will be feasible or useful for more than a few years. A strong argument put forward by those companies to convince people is that, in the future, stem cells might be found very useful. In other words, they are explicit and legally offering hope. 

Using cells collected from deciduous teeth as a source of stem cells represents an incongruity: they do not have a great potential to originate new cells, as they are genetically programmed to die, with structural fragmentation. The genes activated in those cells do not have the potential for regeneration required for them to act as a new source of young cells. Some studies report that cell markers reveal such low potential.^6^

Teeth might and have been used as a source of stem cells for studies, provided that such cells are collected from permanent teeth recently formed or under development - as it is the case of third molars or other permanent teeth subjected to extraction - but not from deciduous teeth. Researches using tissue stem cells extracted from deciduous teeth must have their outcomes thoroughly assessed. Should they be positive, they must be explained on the basis of other principles, not on the basis of a new cell population originating from senescent cells.

Individuals not older than 15-16 years of age have their third molars not yet completely formed; those teeth are indeed dental embryos full of stem cells. Third molars could be extracted between 10 and 12 years of age, and might as well be a rich source of stem cells - unlike deciduous teeth with their cells on the verge of or already under apoptosis! A number of research groups, national and internationally set up, have researched the potential of deciduous teeth-derived stem cells to grow other types of cells, including neurons, namely:

1 - At Instituto Butatantan, located in São Paulo, deciduous teeth-derived stem cells have been employed in human beings by studies^1^ aiming at restoring one's cornea. Nevertheless, they are experimental trials of which outcomes are not expected to reach the overall population in a near future. Those studies began in 2004 and the technique employed to collect and treat deciduous teeth pulp cells allowed them to grow in laboratory. 

2 - At Universidade de São Paulo (USP), a researcher^1^ reported that adult teeth stem cells receded to the induced pluripotent stage (iPs), presenting features similar to those of embryo stem cells. The study was published on *Cell Transplantation* journal and aimed at treating autistic children by means of a noninvasive method. Blood collection and biopsy might be traumatic and effortful for children. iPS skin cells are exposed to light and other environmental contaminants and are, therefore, subject to mutation. Tooth cells, however, remain protected. Nevertheless, the researcher claimed that it is way too soon for outcomes to be extrapolated to human beings.

3 - At Universidade Federal de São Paulo (UNIFESP), researches on third molars considered those teeth as a major source of stem cells capable of growing tissues and regenerating disease-affected body parts. The research was primarily developed at the National Institute of Advanced Industrial Science and Technology in Japan;^5^ however, at UNIFESP, since 2007, researchers experts in Cell Biology and Genetic Therapy were able to transform third molar cells into other cells regenerating bones of the mouth or other body parts.

Convincing parents to store tissue cord blood and/or bone marrow stem cells of their children with hopes of using them in the future is understandable; however, convincing them to keep aged cells under apoptosis, collected from deciduous teeth, and using them as stem cells is a difficult task. Furthermore, it is even more complicated to convince adult patients to keep deciduous teeth with the same purpose.

The media, as well as a few researchers and entrepreneurs, have systematically insisted on deciduous teeth as being a source of stem cells. This is probably a result of lack of in-depth biological knowledge and research on deciduous teeth which, in general, are conducted by non-dental professionals. Change the focus from deciduous teeth to third molars and enjoy data collection!

## FINAL CONSIDERATIONS

Deciduous teeth should not be kept during adulthood, since the consequences are worse than the potential benefits. Cases of partial anodontia of deciduous teeth must be well planned as soon as they have been diagnosed. They require a combination of knowledge and experience shared by pediatric dentists, implant dentists and orthodontists, in addition to other professionals, depending on the specificities of each case. 

Deciduous teeth should not be subjected to orthodontic movement, as this procedure speeds root resorption up. Deciduous teeth should not be considered as an adequate source of stem cells and should not be stored for future therapeutic usage that will rise as potential scientific development. Permanent teeth are rich in undifferentiated cells and have a great potential for regeneration, unlike deciduous teeth cells under apoptosis.
